# Usefulness of Endoscopic Indices in Determination of Disease Activity in Patients with Crohn's Disease

**DOI:** 10.1155/2016/7896478

**Published:** 2016-02-21

**Authors:** Marcin Kucharski, Jacek Karczewski, Dorota Mańkowska-Wierzbicka, Katarzyna Karmelita-Katulska, Elżbieta Kaczmarek, Katarzyna Iwanik, Piotr Rzymski, Marian Grzymisławski, Krzysztof Linke, Agnieszka Dobrowolska

**Affiliations:** ^1^Department of Gastroenterology, Human Nutrition and Internal Diseases, Heliodor Swiecicki Clinical Hospital, Przybyszewskiego 49, 60-355 Poznań, Poland; ^2^Poznan University of Medical Sciences, Fredry 10, 61-701 Poznań, Poland

## Abstract

*Background*. Assessment of endoscopic activity of Crohn's disease (CD) is of growing importance both in clinical practice and in clinical trials. The study aimed to assess which of the endoscopic indices used for evaluation of mucosal changes correlates with the currently used clinical indices for determination of disease activity and with the results of histopathological examination.* Study*. A group of 71 patients with CD and 52 individuals without a diagnosis of GI tract disease as a control group were investigated, considering clinical and histological severity of the disease and the severity of inflammatory changes in the bowel. Evaluation was conducted with the use of clinical, endoscopic, and histopathological indices. Endoscopic indices were then correlated with different clinical and histopathological indices with the aim of finding the strongest correlations.* Results and Conclusions*. Correlation between the clinical disease activity and the severity of endoscopic lesions in CD was shown in this study to be poor. The results also indicate that the optimal endoscopic index used in the diagnostic stage and in the assessment of treatment effects in CD is Simple Endoscopic Score for Crohn's Disease (SES-CD).

## 1. Introduction

Crohn's disease (CD) is a chronic intestinal inflammatory disease characterized by recurrent inflammatory involvement of intestinal segments with several manifestations often resulting in an unpredictable course. CD can involve any portion of the gastrointestinal (GI) tract but most commonly presents with inflammatory changes of the distal small intestine and proximal colon. To date, there are no medications or surgical procedures that can cure Crohn's disease. Treatment options help with symptoms, maintain remission, and can prevent relapse, but even so, about 70% of CD patients undergo surgical resection to treat complications of the disease such as strictures, fistulas, or abscesses [1–3]. Assessment of endoscopic activity of the disease is of growing importance in CD both in clinical practice and in clinical trials. Endoscopy is used for the diagnosis of extent and severity of disease, measurement of treatment effects, treatment delivery, and cancer surveillance. The combination of clinical remission and mucosal healing represents a major goal of the different treatment strategies in CD, especially of biologic and immunomodulatory therapies [4–6]. Complete assessment of severity of a CD patient includes not only macroscopic and microscopic mucosal grading but also physical signs and symptoms, as well as biochemical and hematological criteria.

The aim of the study was to assess which of the endoscopic indices used in CD for evaluation of mucosal changes correlates with the currently used clinical indices for determination of CD activity. It was also the aim to find out which of the studied endoscopic indices correlates with the results of histological examination evaluated in CD patients with the Scoring System for Histological Disease Activity in Crohn's Disease (SSHDACD). The results would indicate the most optimal endoscopic indices for the use in diagnostic workup and evaluation of treatment outcome in CD patients.

## 2. Materials and Methods

### 2.1. Patients

The project was approved by the local ethics committee. The study involved 71 patients with the diagnosis of CD based on colonoscopy with the histological confirmation of the disease with biopsies taken during this endoscopic study or based on histopathological results from surgical material, in this group of patients that required such an intervention prior to the enrollment in the study. All patients included in the study were patients under the care of the Department of Gastroenterology, Human Nutrition and Internal Diseases and assessed at Heliodor Swiecicki Clinical Hospital of Poznan University of Medical Sciences, in the years from 2009 to 2013. Patients included in the CD group were new patients in whom the histopathological diagnosis of UC was made after evaluation of the biopsies taken during colonoscopy on current hospitalization as well as those patients who already had a histopathological diagnosis of CD and were admitted for a planned endoscopic evaluation to check the extent and severity of the disease. All others were excluded from the CD group. Comparison group consisted of 52 individuals who had colonoscopy and agreed for biopsies being taken, prior to the study, for histological examination, but were not diagnosed with IBD or any other diseases of the gastrointestinal tract. The indications for colonoscopy were the need for evaluation of the colon because of symptoms (such as abdominal pain, episodes of diarrhea, and change in bowel movements) but also the need to screen for polyps or neoplasms. Patients before colonoscopy agreed for biopsies to be taken from five intestinal segments: terminal ileum, right colon, transverse colon, left colon, and the rectum. The biopsies were evaluated for microscopic colitis (lymphocytic colitis and collagenous colitis). Endoscopies were performed by two experienced endoscopists from the Department of Gastroenterology, Human Nutrition and Internal Diseases of PUMS. Both endoscopists performed evaluations simultaneously during the procedure and together arrived at a consensus as to the state of the mucosa and findings on endoscopy. The primary endoscopist was performing the colonoscopy on the patient and simultaneously the other endoscopist was also evaluating and assessing the mucosa on the monitor and recording the data when mucosal assessment was made according to particular endoscopic indices used in the study. No videos were taken and assessed by other investigators. The control group was evaluated using clinical indices and endoscopic indices as well as the histological index in the same way as the CD patient group was evaluated in order to make the correlations. If any pathology was seen macroscopically during the colonoscopy by the evaluating endoscopist, patients were not included in the control group. A total of 123 individuals were tested, including 66 women and 57 men. In all of the investigated subjects, subjective assessment, physical examination, and biochemical tests were performed and clinical disease activity was assessed using common clinical activity indices, endoscopic indices, and a histological index (Scoring System for Histological Disease Activity in Crohn's Disease, SSFHDAICD).  Table 1 presents the characteristics as well as biochemical laboratory results for the CD patient group and the control group.

### 2.2. Materials

Material for histological examination consisted of the biopsies collected during the colonoscopic examination from different parts of the colon and small intestine. The study included patients with histologically, radiologically, or surgically earlier confirmed diagnosis of CD. The patients were under the care of the Department of Gastroenterology, Human Nutrition and Internal Diseases and the Gastroenterology Clinic of the Heliodor Swiecicki Clinical Hospital of Poznan University of Medical Sciences during the period from 2009 to 2013. Control group included individuals who had a colonoscopy and agreed to biopsy taking for histological examination but were not diagnosed with inflammatory bowel diseases (IBD) or any other diseases of the GI tract. Material in CD patients was collected from terminal ileum, the right side of the colon (cecum or ascending colon), transverse colon, left side of the colon (descending or sigmoid colon), and the rectum. In the control group, the material was acquired from five intestinal segments as in patients with CD. Several biopsies were taken from each of the intestinal segments so that the material would be representative.

### 2.3. Clinical and Endoscopic Scores

**Figure 1:**
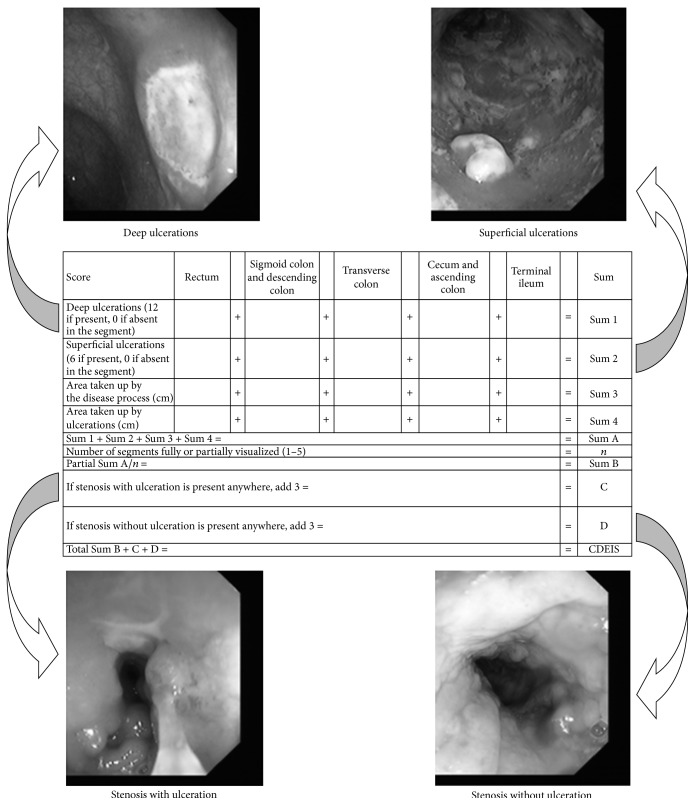
Example of how mucosal changes were evaluated on endoscopy. Crohn's Disease Endoscopic Index of Severity (CDEIS).

In patients with the diagnosis of CD, activity of the disease was calculated in the following five clinical activity indices: Crohn's Disease Activity index (CDAI) [7], Harvey and Bradshaw Index or Simple Index [8], van Hees Index (VHI) or Dutch Index [9], Oxford Index or IOIBD (International Organization for Inflammatory Bowel Disease) [10], and Cape Town Index [11]. In patients with the diagnosis of CD, the extent and severity of disease were assessed in the following endoscopic indices: Crohn's Disease Endoscopic Index of Severity (CDEIS) [12] (Figure 1) and Simple Endoscopic Score for Crohn's Disease (SES-CD) [13]. In CD patients, histological assessment of disease activity in different parts of the large intestine and terminal part of ileum was performed using the Scoring System for Histological Disease Activity in Crohn's Disease (SSFHDAICD) [14], which took into consideration such histological variables as epithelial damage, architectural changes, the presence of mononuclear cells in the lamina propria, polymorphonuclear cells in the lamina propria, the presence of neutrophils in the epithelium, erosions or ulcerations, and the presence of granulomas as well as the number of biopsies affected by the disease process. Patients in the comparison group were also evaluated with the use of the same indices that were used for the evaluation of the CD patient group.

For the purpose of comparison and index correlation, each clinical, endoscopic, and histological index was divided into four categories of disease severity: remission, mild course of the disease, moderate course of the disease, and severe course of the disease.  Table 2 shows how indices used in CD were divided.

### 2.4. Statistical Analysis

The histological, endoscopic, and clinical severity indices in groups analyzed, from the point of view of statistical methods, are ordinal (ranking) indices; therefore, statistical analysis was performed by nonparametric methods. The assessment results in each of the indices in the studied group and the control group are presented in the form of descriptive statistical parameters: mean values, standard deviation, median, and quartiles (Q25 and Q75). Dependencies between endoscopic, histological, and clinical indices assessing severity degree of the changes in a group of CD patients, as well as in the control group, were analyzed using Spearman's ranking correlation coefficient. In addition, for the statistically significant correlation coefficients generally accepted classification of their absolute values was introduced, according to the following criteria [15]: (i) if the correlation coefficient belongs to the 0.00–0.25 range, then the dependency between the studied traits (indices) is negligible (little if any correlation); (ii) if the correlation coefficient belongs to the 0.26–0.49 range, then the correlation of traits is low (low correlation); (iii) if the correlation coefficient belongs to the 0.50–0.69 range, then the correlation is moderate (moderate correlation); (iv) if the correlation coefficient belongs to the 0.70–0.89 range, then the correlation is high (high correlation); (v) if the correlation coefficient belongs to the 0.90–1.00 range, then the correlation is very high (very high correlation). On this basis, statistically significant correlation coefficients, which were greater than or equal to 0.5 (in absolute value), were adopted as results of moderate or high dependency between indices. The results of severity assessment of the clinical, endoscopic, and histological changes in patient group were compared to the control group using Mann-Whitney test. The results were considered significant, if the level of significance *P* ≤ 0.05. Statistical analysis of the results was performed using Statistica v.10 software.

## 3. Results

Characteristics such as age at diagnosis, localization of the disease, and behavior of the disease as well as clinical activity and past surgeries for the CD patient group are shown in Table 3. Characteristics are also shown for the control group studied. In the most widely used clinical index CDAI, 30 patients (42%) had remission of the disease, 21 (30%) patients had mild disease, 16 patients (22%) experienced moderate disease activity, and 4 patients (6%) presented severe disease (CDAI score of >400). Clinical activity of CD was also calculated in four other clinical indices. Averages as well as minimal and maximal values and standard deviations (SD) for the other clinical activity indices were calculated (Table 4).

In the group of CD patients, a correlation of a low coefficient value was demonstrated between the endoscopic index CDEIS and the Harvey and Bradshaw clinical index (*r* = 0.266, Table 5). The rest of the combinations showed a negligible correlation between the endoscopic indices (CDEIS and SES-CD) and all five clinical indices: CDAI, van Hees Index, Harvey and Bradshaw Index, Oxford Index, and Cape Town Index. In this, the highest of them was found between the SES-CD Index and both indices, CDAI and van Hees Index (*r* = 0.235 and *r* = 0.252, resp.), as well as between CDEIS endoscopic index and both CDAI and van Hees Index (*r* = 0.254 and *r* = 0.237, resp.). Negligible correlation was shown between all five clinical indices used for clinical assessment of severity of CD (CDAI, van Hees Index, Harvey and Bradshaw Index, Oxford Index, and Cape Town Index) and histological index (SSFHDAICD). Negligible correlation was also observed in the control group when endoscopic and clinical indices were compared, except that there were low correlations between SES-CD as well as CDEIS and clinical index CDAI (*r* = 0.370 and 0.404, resp.). A low correlation coefficient of 0.325 was also observed between CD histological index and Harvey and Bradshaw clinical index (Table 5).

In the investigated group of CD patients, a significant correlation was demonstrated, according to the criteria used, defined as a moderate correlation, between the endoscopic index SES-CD and histological assessment made using the SSFHDAICD in four of the five intestinal segments from which mucosal biopsies were taken. From all five intestinal segments, the highest correlation was demonstrated in the terminal ileum where CD usually tends to localize. But lower moderate correlations were also obtained in the right side of the colon, the left side of the colon, and the rectum.

In the investigated group of CD patients, a significant correlation was demonstrated, according to the criteria used, defined as a moderate correlation, between the endoscopic index CDEIS and histological assessment made using the SSFHDAICD in two of the five intestinal segments from which mucosal biopsies were taken. Moderate correlations were obtained in the terminal part of the ileum and right side of the colon (Table 6). At the same time, in patients with CD, a moderate correlation was demonstrated in an endoscopic assessment made by the use of SES-CD Index and histological index SSHDACD as well as between CDEIS and histological index, considering in each patient a section of bowel with the most severe endoscopic changes, as well as an area with the most representative inflammatory changes which were rated in a histological index. Correlation was also performed for the people in the control group (Table 6).

The next part of statistical analysis was to present the assessment results of each of the indices in CD patients as well as those from the control group in a form of descriptive statistical parameters: mean values, standard deviation, median, and quartiles (Q25 and Q75). The results of severity assessment of the endoscopic, clinical, and histological indices in CD patients were compared with the control group with a Mann-Whitney test (Table 7).

## 4. Discussion

Endoscopy plays an integral role in the diagnosis, management, and surveillance of IBD. Because there is no single pathognomonic test that establishes the diagnosis of IBD, endoscopy is useful in making the diagnosis, excluding other etiologies, differentiating CD from UC. It also allows defining the patterns, extent, and activity of mucosal inflammation. Endoscopy enables direct visualization of the mucosa and allows for biopsy acquisition and histologic evaluation. In established IBD diagnosis, endoscopy helps to influence clinical and surgical decisions, aids in targeting medical therapies, and allows for the management of IBD related complications. Furthermore, endoscopy plays a key role in the surveillance of patients who are at increased risk for dysplasia and the development of colorectal cancer.

Endoscopic scoring system, CDEIS, that was developed in 1989 in order to assess severity of ileal and colonic disease and validated for monitoring of CD is time consuming and unsuitable for routine endoscopic evaluation of CD patients. CDEIS requires visual analogue scale (VAS) transformations and is too complicated, involving the analysis of multiple aspects of endoscopic lesions. It is based on the presence of four types of lesions: superficial ulcers, deep ulcers, ulcerated stenosis, or stenosis without ulceration, all of which should be recorded in five different segments: terminal ileum, cecum or ascending colon, transverse colon, descending and sigmoid colon, and the rectum [13]. After endoscopic evaluation, assessment, and value recording, calculation is required to receive the severity score, which ranges from 0 to 44. This process is time consuming and not practical in the clinical setting. The differentiation between deep and superficial ulcers is difficult during endoscopy whereas size might be more important in this index. That same group that developed it, the GETAID group (Groupe d'Etudes Therapeutiques des Affections Inflammatoires Digestives), demonstrated that the use of endoscopy and CDEIS to guide therapeutic decisions with regard to corticosteroid therapy was not helpful [17].

The calculations in SES-CD are easier and can be done faster than in CDEIS and the reproducibility of parameters was confirmed. The index was derived from the CDEIS; it is therefore also highly correlated with CDEIS. SES-CD is reliable, reproducible, and very easy to use. It includes only relevant endoscopic lesions and avoids complicated measurements and conversions by using numerical values instead of visual analogue scale and therefore it allows for the score to be easily calculated by the endoscopist on-site [13, 14].

While conducting this research, it was demonstrated that SES-CD and CDEIS, the two endoscopic indices most commonly used for assessment of mucosal lesions, moderately correlate with CD histological index (SSFHDAICD). SES-CD endoscopic index had a better result in the correlation because the correlation was moderate in four of the five sections from which biopsies were taken (terminal ileum, right colon, left colon, and the rectum). The highest of them was the one in the terminal part of the ileum, typical localization for CD. In CDEIS, the moderate correlation occurred only in two out of the five sections of the intestine studied (terminal ileum and right colon). Taking into consideration other studies which show close correlation of CDEIS (gold standard) and SES-CD, as well as demonstrating that, in clinical routine, the SES-CD is easier to use and could replace the CDEIS [18], it should be even further said that because SES-CD correlates better than CDEIS with histopathology results it should be rather used in the clinical practice instead of CDEIS, even though the correlation of both of the endoscopic indices and the five clinical indices evaluated in this study is negligible (little if any correlation). Poor correlation of endoscopic indices and clinical indices has been also reported previously by other investigators.

Although the above conclusions can be made, there are some evident limitations of endoscopic scores of bowel lesions in CD. For one, the mucosal activity does not always reflect transmural damage and such complications of the disease as fistulas and abscesses (e.g., perianal, intra-abdominal) are not endoscopically evaluated and are not scored in any of the above indices. Another shortfall of the endoscopic indices is that there are still no validated endpoints for endoscopic response, endoscopic remission, or validated definition of complete mucosal healing. As Dave and Loftus state in their paper, “currently, there is no validated definition of what constitutes mucosal healing in inflammatory bowel disease” [19].

In recent years, mucosal healing is gaining more acceptance as a measure of disease treatment effectiveness and is also becoming the desired endpoint in clinical trials. Why is mucosal healing important as the goal of IBD treatment? The main hypothesis in support of mucosal healing is that achieving it may improve quality of life, prevent IBD relapse, minimize hospitalization, and alter the natural history of the disease and it can minimize the lifetime risk for surgical interventions. Mucosal healing is a more objective endpoint than clinical remission for evaluating inflammatory disease activity and should be used in both therapeutic trials and clinical practice for the management of IBD patients. Prior publications have correlated achievement of mucosal healing with good end-treatment outcomes. In CD, mucosal healing has been achieved with corticosteroids, enteral nutrition (in pediatric patients), immunosuppressive drugs, infliximab, and adalimumab and has been maintained with immunosuppressive drugs and biological agents [20–22].

There are few indices to assess endoscopic disease activity in CD. Physicians need to remember this when comparing the rates of mucosal healing across studies, since minor changes in the definition of mucosal healing or the use of indices with just slight changes to their variables may result in considerable differences in healing rates presented.

Mucosal healing as assessed by endoscopy is a useful tool evaluating and guiding response to therapy in patients with IBD. However, performing endoscopy on a frequent, regular basis may have drawbacks. Patients discomfort and compliance are both issues. Also, while generally considered to be a low-risk procedure, diagnostic endoscopy still carries risk of perforation and bleeding and cardiovascular risks due to anesthesia, particularly in patients with IBD. In addition, endoscopy is an expensive procedure, and frequent endoscopy may not prove to be cost-effective. Finally, colonoscopy without biopsies may not be able to completely assess treatment response and may not diagnose early neoplastic lesions or predict long-term outcomes such as hospitalization or surgical procedures.

Newer clinical trials are incorporating mucosal healing as an endpoint for evaluation of efficacy. However, we do not yet have prospective trials demonstrating that escalation of therapy to achieve mucosal healing alters long-term outcomes for patients. ECCO consensus conference on mucosal healing in IBD concluded that mucosal healing is important, but this conference highlighted the need for large, prospective studies assessing the impact of mucosal healing on the natural course of the disease. The use of different indices by endoscopists for endoscopic assessment does not help the researchers [23].

Currently, as this study shows, the best measure for evaluation of mucosal changes in CD is the Simple Endoscopic Score for CD (SES-CD). That is why this index should be used in each colonoscopic procedure performed by an endoscopist evaluating the mucosa of a patient with CD. This is crucial for patient follow-up and modification of treatment in time to change the natural course of the disease. This will standardize the presentation assessment and help to guide patients in their treatment.

In a study of patients with long-standing CD, postinflammatory polyps as well as strictures on endoscopy were associated with a significantly increased cancer risk [24]. One thing that can be said for sure is that IBD definitely increases the risk of colon cancer and long-standing inflammation (chronic inflammation) even more so. Endoscopic changes seen macroscopically should be monitored for improvement and response to treatment with endoscopic indices. But one of the major difficulties in identifying dysplastic mucosa during colonoscopy arises in that the majority of changes occur within macroscopically normal tissue. As a result, the accuracy in predicting dysplasia correlates with the number of biopsies taken. It has been estimated that, to exclude dysplasia with a 90% certainty, 33 biopsy specimens are required, and to increase the accuracy to 95%, nearly twice the number of biopsy specimens is required [25]. This is probably why in the analysis of research for this study moderate correlations were obtained between endoscopic indices and histological ones in CD. We are not always able to take a biopsy from the site that we see macroscopically changed in endoscopic study; sometimes changes in close vicinity are biopsied and histological picture from those sites can be totally different. Even if the proper place is biopsied, it might not be enough material; the material might be damaged by the biopsy procedure itself or during preparation. Therefore, the number of biopsies is crucial in showing the actual state of the disease process. The more the biopsies taken, the better the chance of diagnosis of the sites histologically most severely changed by inflammatory bowel disease. Current surveillance strategies call to minimize clinical relapse periods, annual colonoscopy, with multiple biopsies (4 circumferential) taken at every 10 cm intervals, with additional biopsies taken from sites of strictures or raised lesions.

Transmural disease is an important and unique feature of CD. Colonoscopy with biopsy is not a procedure for the diagnosis of transmural disease. Endoscopy also is an invasive technique that is difficult to repeat in patients. However, this feature can be evaluated with a computer tomography (CT) scan or magnetic resonance imaging (MRI) assessing the disruptive layered structure of bowel wall. An interesting study has shown that the magnitude of various quantitative MRI changes, such as wall thickening, contrast signal intensity, and relative contrast enhancement, parallels the severity of endoscopic lesions in Crohn's disease. This finding has enabled the definition of magnetic resonance activity index (MRAI) for CD activity that correlates well with the CDEIS [26]. Additionally, endoscopic ultrasound (EUS) has been used to assess transmural disease [27–29]. However, EUS has not become a standard of care to assess the patient with CD. CD causes asymmetric wall thickening. In patients with penetrating type of CD with intra-abdominal fistulas or perianal fistulizing disease, endoscopic assessment might not be as important as in patients who present nonpenetrating and nonstenotic disease or have just luminal stenotic disease with characteristic mucosal changes. This aspect shows a shortfall in the endoscopic assessment. Endoscopic indices used in CD do not take into consideration such changes and they might not be visible on endoscopy, but they are very important. Endoscopic index score in such a patient will be low and is not representative of the disease severity. Therefore, in the diagnosis and treatment, all available information from history taking, physical exam, and biochemical and radiological studies, as well as other diagnostic modalities, has to be taken into consideration. Endoscopy alone in a good number of cases is not enough. In such patients depending on the type of the disease or its localization, other indices can be used such as Irving Score [30] or Perianal Crohn's Disease Activity Index [31] for patients with perianal disease to assess severity of the disease process and its complications. In patients with the disease localized in the small intestine nonaccessible to standard endoscopy (colonoscopy, gastroscopy), the use of capsule endoscopy and available Capsule Endoscopy Scoring Index [32] can help in the assessment of disease activity and in making therapeutic decisions. Capsule endoscopy has been shown to be more sensitive in the diagnosis of small bowel CD than barium follow-through and computed tomography enteroclysis [33, 34]. These indices were not analyzed in this study but their importance and usefulness should be investigated in subsequent research.

As seen in this study, endoscopy and histology are so crucial in the diagnosis, treatment, and follow-up of IBD patients; the initiation of the use of both of them simultaneously was inevitable. Recent advances in endoscopic imaging techniques have revolutionized the diagnostic approach in patients with IBD. Several new emerging endoscopic imaging techniques can visualize new mucosal details even at the cellular and subcellular level. Such techniques include chromoendoscopy, magnification endoscopy, confocal spectroscopy, laser endomicroscopy, and endocytoscopy [35]. Maybe in the near future these modern endoscopic techniques will be widely available and will make the diagnosis and management of patients with IBD much easier. For now, white light endoscopy remains the gold standard.

Endoscopy in recent years is gaining competition from the ongoing process of development of reliable fecal markers to assess inflammation in IBD. As they are derived from stool, they are of easy access. They may also have a higher specificity than serum markers, since they may reflect intestinal rather than systemic inflammation, a result of the close contact of stools with intestinal mucosa and of the possibility that it may wash out molecules related to inflammation or damage [36, 37]. Finally, fecal markers are gaining popularity because they may cause patients to avoid having to go through frequent endoscopies, since these markers are related to mucosal inflammation and might be able to help assess disease activity in the intestine [38, 39]. The performance of the fecal markers lactoferrin, PMN elastase, and calprotectin, along with CRP and clinical indices, compared to endoscopic measures of inflammation has been evaluated. The three fecal markers are able to define disease activity both in UC and in CD and distinguish IBD from IBS in some situations depending on the marker, even in the absence of disease activity. None of the three markers seem to be superior in their ability to reflect endoscopic inflammation, but all three are superior to CRP in their diagnostic accuracy [40]. Calprotectin can be used in disease monitoring, showing a closer correlation to endoscopic and histological evidence of inflammation than clinical indices and confirming inflammatory activity before the manifestation of clinical signs [41, 42]. However, calprotectin seems to be more predictive of relapse in UC than in CD [43]. Fecal calprotectin was shown also to be a useful screening tool for identifying patients who are most likely to need endoscopy for suspected IBD [44]. But for now endoscopic evaluation and assessment of inflammatory mucosal changes with a naked eye remains irreplaceable.

According to the results of tests performed during this study, it can be said that currently the most optimal endoscopic index used in the diagnostic stage as well as in the assessment of treatment effects in CD is the SES-CD. SES-CD correlates moderately with histological index (Scoring System for Crohn's Disease) in four out of the five studied intestinal segments (CDEIS only correlates in two segments) and to an equal measure as the CDEIS (Crohn's Disease Endoscopic Index of Severity) correlates with two of the clinical indices, CDAI and van Hees Index. Almost all publications also point to the fact that there is no good correlation between endoscopy and clinical assessment in CD patients. SES-CD is also much simpler to use, reliable, and reproducible. It includes only relevant endoscopic lesions and avoids complicated measurements and conversions by using numerical values. Because of these assets, SES-CD should soon replace the CDEIS both in everyday practice and in clinical trials.

Little or no correlation was also found during this study between the five clinical indices studied and histological index, Scoring System for CD. This poor correlation between the clinical aspect of the disease and the histologically evaluated intestinal biopsies points to the need for more frequent histological evaluation because clinically inactive disease does not mean absence of inflammatory activity. It also points to the importance of surveillance because even in clinically inactive disease neoplastic transformation can take place with active mucosal inflammation especially in a patient with long-standing disease process. Further studies are needed to develop standardized endoscopic scoring systems for mucosal healing that will be validated in prospective clinical trials evaluating long-term outcomes for CD. When this is done, only the validated endoscopic indices will be used in clinical practice by physicians and by research professionals.

## Figures and Tables

**Table 1:** Average values for age, body mass, height, BMI, number of daily stools, body temperature, and pulse as well as biochemical laboratory results for the CD patient group and the control group during hospitalization.

Variable [units]	CD patient group				Control group			
	Average	Minimum	Maximum	SD	Average	Minimum	Maximum	SD
Age [years]	34.55	18.0	70.0	12.99	49.35	23.0	82.0	17.92
Body mass [kg]	62.07	36.0	90.0	13.73	70.92	42.0	110.0	16.70
Height [cm]	169.39	147.0	187.0	10.35	168.22	153.0	188.0	8.39
BMI [kg/m^2^]	21.60	12.8	32.0	3.46	25.01	16.0	43.0	5.31
Number of stools/24 h	4.29	1.0	15.0	3.50	2.42	1.0	12.0	2.11
Temp. [°C]	36.75	36.2	39.0	0.54	36.48	36.2	37.6	0.30
Pulse [beats/min]	77.52	60.0	100.0	5.95	75.67	60.0	94.0	4.61
ESR [mm/h]	35.10	3.0	138.0	26.14	14.38	2.0	83.0	15.27
CRP [mg/L]	31.21	0.2	217.5	41.49	4.51	0.2	52.9	8.84
WBC [×10^3^/*μ*L]	7.58	2.7	21.0	3.55	6.42	3.5	11.5	1.85
RBC [×10^6^/*μ*L]	4.34	2.5	5.7	0.68	4.81	3.4	12.7	1.31
Hgb [g/dL]	12.47	6.7	17.5	2.08	14.07	10.4	17.3	1.76
Hct [%]	37.48	22.3	50.2	5.55	40.81	32.1	49.0	4.34
Total protein [g/dL]	6.95	5.3	8.0	0.65	7.23	4.7	8.6	0.76
Albumin [g/dL]	3.98	2.2	5.0	0.61	4.65	2.7	5.7	0.51
Na^+^ [mmol/L]	138.87	131.0	146.0	2.63	139.58	131.0	146.0	2.88
K^+^ [mmol/L]	4.29	2.9	5.8	0.48	4.30	3.4	5.8	0.47
Fe [*μ*g/dL]	65.00	8.0	230.0	49.12	115.67	32.0	243.0	48.26
TIBC [*μ*g/dL]	301.04	126.0	519.0	87.56	306.69	65.0	480.0	76.10
Urea [mg/dL]	23.61	7.0	96.0	12.60	29.61	15.0	81.0	13.18
Creatinine [mg/dL]	0.77	0.4	2.9	0.32	0.96	0.5	7.5	0.96

CRP, C-reactive protein; RBC, red blood cells; UC, ulcerative colitis; WBC, white blood cells; TIBC, total iron binding capacity; Hgb, Hemoglobin; Hct, hematocrit; ESR, erythrocyte sedimentation rate.

**Table 2:** Division of clinical, endoscopic, and histological indices into four CD disease activity categories for comparison purposes.

	Remission	Mild disease	Moderate disease	Severe disease
Clinical indices				
*Clinical index (range)*				
CDAI (0–>400)	<150	150–219	220–400	>400
van Hees Index	<100	100–150	151–210	>210
Harvey and Bradshaw Index	<5	5–7	8–16	>16
Oxford Index	<2	2–4	5–7	8–10
Cape Town Index	1–3	4–10	11–20	21–30

Endoscopic indices				
*Endoscopic index (range)*				
CDEIS (0–44)	<3	3–9	10–12	>12
SES-CD (0–60)	0–2	3–6	7–15	>15

Histological indices				
*Histological index (range)*				
SSFHDAICD (0–16)	0–4	5–7	8–10	11–16

CDEIS: Crohn's Disease Endoscopic Index of Severity; SES-CD: Simple Endoscopic Score for Crohn's Disease; SSFHDAICD: Scoring System for Histological Disease Activity in Crohn's Disease.

**Table 3:** Characteristics of the studied CD patient group and control group, for which assessments were performed in clinical, endoscopic, and histological indexes.

Variable	CD patients	Control group
Number of people evaluated	71	52
Age range	18–70	23–82
Average age	34.5 ± 13	49.3 ± 17.9
Gender, F/M (%)	37/34 (52/48%)	29/23 (56/44%)
BMI	21.6 ± 3.5	25.0 ± 5.3
CDAI	25–488 ± 108.6	—
Remission: CDAI <150	30 (42%)	—
Mild: CDAI 150–219	21 (30%)	—
Moderate: CDAI 220–400	16 (22%)	—
Severe: CDAI >400	4 (6%)	—
Montreal Classification of CD [16]		
Age at diagnosis		
A_1_: <17 years old	8	—
A_2_: between 18 and 40 years old	50	—
A_3_: above 40 years old	13	—
Location		
L_1_: ileal	8	—
L_2_: colonic	19	—
L_3_: ileocolonic	44	—
L_4_: isolated upper GI disease	1	—
Behavior		
B_1_: nonstricturing, nonpenetrating	13	—
B_2_: stricturing	25	—
B_3_: penetrating	33	—
p: perianal disease modifier	10	—
Past surgeries		
Appendectomy	7	—
Partial resection of small intestine	14	—
Partial resection of the colon	12	—
Fistula operations	12	—
Presence of abscess	5	—
Presence of anal fissure	3	—

**Table 4:** Averages as well as minimal and maximal values and standard deviations (SD) for the calculated clinical activity indices in CD patient group and the control group.

Activity index	Average	Minimum	Maximum	SD
Crohn's disease				
CDAI	188.21	25	488	108.64
van Hees	146.71	28.3	257.7	51.77
Harvey and Bradshaw	8.65	3	23	4.60
Oxford	3.70	0	9	1.82
Cape Town	8.41	1	23	4.62

Control group				
CDAI	106.52	3	283	59.52
van Hees	87.08	33.9	232.8	35.74
Harvey and Bradshaw	4.60	1	22	3.36
Oxford	1.50	0	4	1.04
Cape Town	2.52	0	9	1.98

**Table 5:** Correlations of results for evaluation by clinical indices (Crohn's Disease Activity Index, van Hees Index, Harvey and Bradshaw Index, Oxford Index, and Cape Town Index) and two endoscopic indices (SES-CD and CDEIS) for patients with Crohn's disease and control group (correlation was made between the five mentioned clinical indices and the most representative endoscopic segment). The same five clinical indices were correlated with histological index (Scoring System for Histological Disease Activity in Crohn's Disease) (correlation was made between the five mentioned clinical indices and the most representative section in the histological index).

		Clinical indices				
		CDAI	van Hees Index	Harvey and Bradshaw Index	Oxford Index	Cape Town Index
CD patient group						
Endoscopic index						
SES-CD global		0.235	0.252	0.204	0.082	0.201
CDEIS global		0.254	0.237	0.267	0.148	0.223

Histological index	Hist. score maximum	0.139	0.103	0.160	−0.001	0.112

Control group						
Endoscopic index						
SES-CD global		0.370	−0.025	0.033	0.214	0.247
CDEIS global		0.404	0.001	0.052	0.248	0.245

Histological index	Hist. score maximum	0.029	0.128	0.325	0.207	0.222

**Table 6:** Correlations of results for evaluation by histological index (Scoring System for Crohn's Disease) and two endoscopic indices (Simple Endoscopic Score for Crohn's Disease and Crohn's Disease Endoscopic Index of Severity) for patients with Crohn's disease and the control group. Correlations made in five intestinal segments: terminal ileum (ILE), right colon (RCO), transverse colon (TRA), left colon (LCO), and rectum (REC).

	Hist. ILE	Hist. RCO	Hist. TRA	Hist. LCO	Hist. REC
Crohn's disease					
SES-CD ILE	**0.598**	0.384	0.200	0.079	0.212
SES-CD RCO	0.126	**0.508**	0.384	0.176	0.381
SES-CD TRA	0.050	0.385	0.451	0.238	0.258
SES-CD LCO	0.036	0.319	0.472	**0.508**	0.534
SES-CD REC	0.136	0.239	0.383	0.357	**0.550**

CDEIS ILE	**0.558**	0.450	0.306	0.172	0.277
CDEIS RCO	0.254	**0.564**	0.401	0.128	0.323
CDEIS TRA	0.118	0.401	0.451	0.241	0.270
CDEIS LCO	0.058	0.259	0.420	0.432	0.513
CDEIS REC	0.111	0.151	0.300	0.286	0.484

	Hist., most severe				

SES-CD global	**0.600**				
CDEIS global	**0.554**				

Control					
SES-CD ILE	0.374	−0.212	−0.211	−0.233	−0.232
SES-CD RCO	0.142	0.215	0.165	0.183	0.042
SES-CD TRA	0.341	0.202	0.333	0.169	0.124
SES-CD LCO	0.282	0.089	0.100	0.365	0.075
SES-CD REC	0.282	0.089	0.090	0.310	0.341

CDEIS ILE	0.374	−0.201	−0.200	−0.220	−0.220
CDEIS RCO	0.091	0.215	0.170	0.183	0.042
CDEIS TRA	0.345	0.216	0.345	0.190	0.143
CDEIS LCO	0.288	0.120	0.134	0.387	0.113
CDEIS REC	0.288	0.113	0.118	0.336	0.369

	Hist., most severe				

SES-CD global	0.135				
CDEIS global	0.110				

Values in bold show moderate correlation (correlation coefficient in the 0.50–0.69 range).

**Table 7:** Assessment results in each of the indices in CD patients as well as in the control group presented in the form of descriptive statistical parameters: mean values, standard deviation, median, quartiles (Q25 and Q75), and Mann-Whitney (MW) test.

Group	Mean	SD	Q25	Median	Q75	MW test
SES-CD whole						
CD (*n* = 71)	8.55	7.51	3.00	7.00	13.00	*P* < 0.0001
Control (*n* = 52)	1.31	1.74	0.00	1.00	2.00	

CDEIS whole						
CD (*n* = 71)	9.70	9.11	2.50	7.20	14.00	*P* < 0.0001
Control (*n* = 52)	2.81	13.99	0.00	0.25	1.00	

CDAI						
CD (*n* = 71)	188.21	108.65	107.00	164.00	241.00	*P* < 0.0001
Control (*n* = 52)	106.52	59.52	57.00	105.50	142.50	

van Hees Index						
CD (*n* = 71)	146.71	51.78	110.94	139.00	179.61	*P* < 0.0001
Control (*n* = 52)	87.08	35.74	64.06	77.63	107.13	

Harvey and Bradshaw Index						
CD (*n* = 71)	8.65	4.60	5.00	8.00	11.00	*P* < 0.0001
Control (*n* = 52)	4.60	3.36	2.50	4.00	5.00	

Oxford Index						
CD (*n* = 71)	3.70	1.82	3.00	4.00	5.00	*P* < 0.0001
Control (*n* = 52)	1.50	1.04	1.00	1.50	2.00	

Cape Town Index						
CD (*n* = 71)	8.41	4.62	5.00	8.00	11.00	*P* < 0.0001
Control (*n* = 52)	2.52	1.98	1.00	2.00	3.00	
